# A cFos activation map of remote fear memory attenuation

**DOI:** 10.1007/s00213-018-5000-y

**Published:** 2018-08-17

**Authors:** Bianca A. Silva, Allison M. Burns, Johannes Gräff

**Affiliations:** Laboratory of Neuroepigenetics, Brain Mind Institute, School of Life Sciences, École Polytechnique Fédérale Lausanne, 1015 Lausanne, Switzerland

**Keywords:** Remote memory, Extinction, cFos, PTSD, Contextual fear conditioning, Neuronal network, Cortex, Hippocampus, Thalamus, Amygdala

## Abstract

**Rationale:**

The experience of strong traumata leads to the formation of enduring fear memories that may degenerate into post-traumatic stress disorder. One of the most successful treatments for this condition consists of extinction training during which the repeated exposure to trauma-inducing stimuli in a safe environment results in an attenuation of the fearful component of trauma-related memories. While numerous studies have investigated the neural substrates of recent (e.g., 1-day-old) fear memory attenuation, much less is known about the neural networks mediating the attenuation of remote (e.g., 30-day-old) fear memories. Since extinction training becomes less effective when applied long after the original encoding of the traumatic memory, this represents an important gap in memory research.

**Objectives:**

Here, we aimed to generate a comprehensive map of brain activation upon effective remote fear memory attenuation in the mouse.

**Methods:**

We developed an efficient extinction training paradigm for 1-month-old contextual fear memory attenuation and performed cFos immunohistochemistry and network connectivity analyses on a set of cortical, amygdalar, thalamic, and hippocampal regions.

**Results:**

Remote fear memory attenuation induced cFos in the prelimbic cortex, the basolateral amygdala, the nucleus reuniens of the thalamus, and the ventral fields of the hippocampal CA1 and CA3. All these structures were equally recruited by remote fear memory recall, but not by the recall of a familiar neutral context.

**Conclusion:**

These results suggest that progressive fear attenuation mediated by repetitive exposure is accompanied by sustained neuronal activation and not reverted to a pre-conditioning brain state. These findings contribute to the identification of brain areas as targets for therapeutic approaches against traumatic memories.

## Introduction

Traumatic events induce some of the most persistent forms of memory, which contribute to the pathogenesis of a number of stress and anxiety-related disorders including post-traumatic stress disorder (PTSD). One of the most efficient treatments for these conditions is known as exposure therapy and consists of the repeated re-exposure to fear-inducing stimuli in a safe environment. This in turn leads to the attenuation of the pathological fear component associated to the traumatic memory (Foa and Kozak 1986). Since this and other interventions are often not available immediately following the traumatic event, the identification of effective attenuation protocols and the underlying neural mechanisms for the attenuation of remote traumatic memories are of primary importance.

In rodents, fear memory attenuation is modeled by extinction paradigms. Briefly, animals are subjected to fear conditioning during which an unconditioned noxious stimulus (US) is paired with a neutral conditioned stimulus (CS). After this association has been formed, the presentation of the CS alone induces high fear responses (memory recall); however, repeated exposure to the CS mediates a progressive reduction of fear responses (fear extinction). Such paradigms have been widely exploited to investigate the neural circuits at the basis of 1-day-old fear memory extinction (for review, see Bouton 2004; Myers and Davis 2007; Herry et al. 2010). However, considerably less is known about the neural circuits mediating remote (1-month-old) fear memory extinction. Indeed, extinction procedures lose their efficacy as time increases between the first encoding of the traumatic memory and its treatment (see Tsai and Gräff 2014 for a review).

Extinction processes are tightly connected with memory retrieval (Tronson and Taylor 2007; Costanzi et al. 2011; Inda et al. 2011; An et al. 2018), the neuroanatomical correlates of which are thought to shift with memory age (Frankland and Bontempi 2005; Lopez et al. 2008; Corcoran et al. 2013; Gräff et al. 2014; Albo and Gräff 2018). In particular, memory retrieval has been shown to become progressively less dependent on brain structures involved in memory encoding, such as the hippocampus, and more dependent on alternative substrates, which are thought to reside in a more widely distributed cortical network (Frankland and Bontempi 2005; Wheeler et al. 2013). Therefore, it is possible that the temporal shift in the circuits mediating memory retrieval is mirrored by an analogous shift in extinction circuits. For example, unlike the infralimbic cortex (IL), which is involved during both recent and remote extinction (Vetere et al. 2012; Rosas-Vidal et al. 2014; Awad et al. 2015) molecular adaptations in the retrosplenial cortex are only necessary for remote extinction (Corcoran et al. 2013).

Despite such evidence, a comprehensive map of the brain structures recruited by remote fear memory extinction, as compared to remote fear memory recall, is still missing. To address this question, we designed an efficient extinction protocol for attenuating remote contextual fear memories in the mouse and subsequently generated an activity map in selected cortical, thalamic, amygdalar, and hippocampal structures by means of cFos expression, an immediate early gene widely used for brain activity mapping (Guzowski et al. 2005). Based on these data, we computed inter-regional correlations of cFos activity and generated a functional connectivity network map for remote fear memory attenuation and recall.

## Materials and methods

### Animals

Animals used were C57BL/6JRj male mice obtained from Janvier Labs, France. Animals were delivered at 7 weeks of age and allowed an acclimatization period of 1 week before behavioral testing. All animals were housed in groups of four animals at 22–25 °C on a 12 h light-dark cycle (light on 7 AM) with water and food ad libitum. All animals were handled according to protocols approved by the Swiss animal license VD-2808 and VD-2808.1.

### Behavioral testing

Animals were subjected to contextual fear conditioning consisting of 3 min habituation (baseline, BL) to the conditioning chamber (MultiConditioning System, TSE systems GmbH) followed by three 2 s foot shocks (0.8 mA) with an interval of 28 s. After the shocks, the animals were kept in the conditioning chamber for an additional 15 s. Animals belonging to the “Context Only” group underwent the same procedure but did not receive the foot shocks. Four weeks later, mice were re-exposed to the same chamber for 3 min without receiving the foot shock (“Recall”) and returned to their home cage. On the following day, they were re-exposed to the same context two times for 3 min each, separated by a 2 h interval, during which they were returned to their home cage. The same procedure was repeated for 4 days. One day later, mice were re-exposed to the fear conditioning context for an additional 3 min to test their extinction memory (EM). Two weeks later, the spontaneous recovery (SR) of the extinguished memory was assessed by testing freezing during a 3 min exposure to the conditioning context. Animals used for histological analysis were sacrificed 90 min after the last extinction session of a spaced extinction paradigm (“Extinction” and “Context Only” groups) or 90 min after the first conditioned context re-exposure (“Recall” group). In addition to the “Extinction,” “Recall,” and “Context only” groups, a “Home cage” group was used, which was subjected to the whole spaced extinction behavioral paradigm and sacrificed 24 h after the last extinction session in order to account for baseline cFos activity. All behavioral testing was performed between 8 AM and 12 AM and animals were randomly assigned to the different experimental groups. Percentage of time spent freezing over total context exposure time was automatically calculated with an infrared beam detection system (MultiConditioning System, TSE systems GmbH). Freezing was quantified when absence of movement was detected for more than 2.5 s.

### Histology

For cFos immunohistochemistry (IHC), mice were deeply anesthetized with pentobarbital (150 mg/kg intraperitoneally, Streuli Pharma, Switzerland) and perfused trans-cardially (4.0% paraformaldehyde, 1X PBS, pH 7.4). Brains were removed, post-fixed (4% PFA overnight), and cryoprotected (30% sucrose, 1X PBS, 4 °C, 48 h). They were then frozen and 40 μm coronal sections were cut with a sliding cryostat (Leica Microsystems, Germany).

Subsequently, free floating sections were incubated in blocking solution (1% BSA, 1X PBS, 0.3% TrytonX100) at room temperature for 1 h, followed by incubation with rabbit anti-cFos antibody (1:5000, Synaptic System, Germany, #226 003) in blocking buffer (1% BSA, 1X PBS, 0.1% TrytonX100) overnight at 4 °C under constant shaking. Sections were washed extensively with PBS Tryton 0.1% and then exposed to the secondary antibody (Alexa Fluor 647-conjugated donkey anti-rabbit IgG, Life Technologies, USA) in blocking buffer at room temperature for 2 h. After extensive washing, the sections were incubated with Hoechst (Life Technologies, USA) at 1:1000 in PBS at room temperature for 5 min. Slices were washed extensively with PBS and mounted on superfrost glass slides (ThermoScientific, USA) with Fluoromount mounting medium (SouthernBiotech, USA). Images were acquired on a virtual slide microscope (VS120, Olympus, Japan) with a 10 × objective.

### Image analysis

For the detection of cFos-positive cells, images were analyzed with QuPath v0.1.2 (Bankhead et al. 2017). Briefly, brain areas were manually outlined based on the Hoechst signal following the Allen Brain Reference Atlas and cFos-positive cells within the outlined structures were automatically detected with the “positive cell detection” built-in QuPath function. The density of cFos-positive cells (cFos+/mm^2^) was averaged over 2–6 sections per animal.

## Statistical analysis

Data analysis was performed with PRISM 7 software (GraphPad, San Diego, CA). All data are reported as mean ± standard error measurement. For the initial spaced extinction behavioral analysis, statistical significance was determined by one-way ANOVA; for behavioral analysis of the four groups further used for cFos activation, statistical significance was calculated by two-way ANOVA. Statistically significant ANOVA analyses were followed by Holm-Sidak post-hoc multiple comparison analysis. Statistical significance of cFos quantifications was determined by one-way ANOVA of cFos density after home cage, context only, recall, and extinction followed by Sidak post-hoc multiple comparison analysis in case of significance.

### Inter-regional correlation analysis

Within each experimental group (“Home cage,” “Context Only,” “Recall,” “Extinction”), Pearson correlation coefficients (Harrel et al. 2018) were calculated for the pairwise comparisons of cFos density between all 16 brain regions analyzed. Brain region correlations reflecting fewer than four pairs were excluded for all subsequent visualization and analysis. Correlations were displayed as a color-coded correlation matrix using a custom R-code (R version 3.4.4) (R Development Core Team 2011).

### Network connectivity and correlation analysis

Network comparisons were constructed using both cFos density calculations and Pearson correlation coefficients. Each node represents one of the 16 brain regions examined in this study. Node sizes are proportional to the cFos density increase from each brain region in each respective experiment compared to the cFos densities in the “Home cage” experiment. The network connection lines represent Pearson correlations between brain regions and were filtered to represent correlations that were calculated using four or more pairs and had a *P* value ≤ 0.1 and an *r* value ≥ 0.5. Line transparency relates to the *r* value of the correlation of the two regions with black being more correlated and white being less correlated. The igraph package (v1.2.1) (Csárdi and Nepusz 2006) in R was used to visualize the networks. Negative correlations are not represented in the network maps.

## Results

### An extinction paradigm to attenuate remote fear memory

**Fig. 1:**
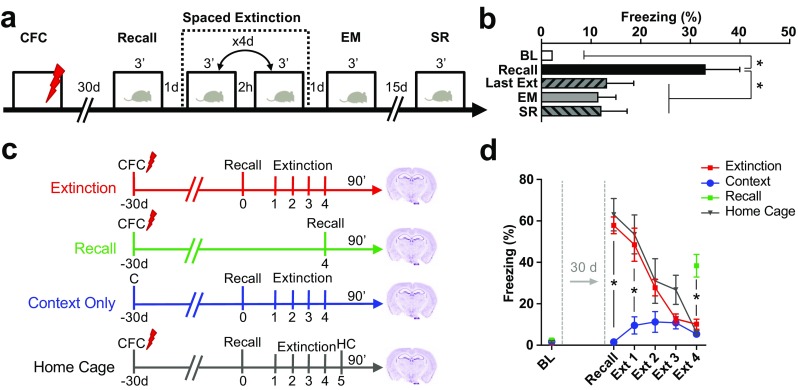
Remote fear memory is efficiently attenuated by a spaced extinction paradigm. **a** Schematic representation of the experimental setting. Mice underwent contextual fear conditioning (CFC) and were re-exposed to the conditioned context 30 days later (Recall). On the following day, the animals were subjected to spaced extinction where they were re-exposed twice per day for 4 days to the conditioned context in the absence of foot shock. One and 15 days later, both groups received an additional context exposure to test their extinction memory (EM) and spontaneous recovery (SR). **b** Freezing levels across the spaced extinction procedure. During recall, freezing was significantly increased compared to baseline (BL, 3 min context exposure before conditioning), and during the last extinction session, extinction memory and spontaneous recovery freezing was significantly decreased compared to recall (ANOVA, *F*(4, 51) = 6.76, *P* = 0.0002, *n* = 8–16). **c** Experimental design for cFos analysis. **d** Freezing levels of all experimental animals further used for cFos analysis. At remote recall and during the first extinction sessions, freezing was significantly increased in animals that received the foot shock (“Home Cage,” “Recall,” and “Extinction”) compared to animals that received no shocks in the conditioning session (“Context Only”). At the end of extinction, animals that did or did not receive the foot shock in the conditioning context showed no significant differences in freezing (two-way ANOVA, *F*(1, 24) = 33.3, *P* < 0.0001, *n* = 10–16 per group). *P < 0.05 by Holm-Sidak post-hoc test

In order to assess brain activation patterns after remote fear memory attenuation, we established a behavioral paradigm that could efficiently and persistently reduce freezing to the conditioned context 1 month after contextual fear conditioning. Remote memory, as opposed to recent memory, has been shown to be more resistant to attenuation (Costanzi et al. 2011; Gräff et al. 2014) and to strongly depend on the type and duration of the extinction protocols used (Lopez et al. 2008; Costanzi et al. 2011; Inda et al. 2011). For this reason, we designed a spaced extinction behavioral paradigm preceded by an isolated recall session. Animals were first subjected to contextual fear conditioning during which, after 3 min of habituation to the novel context (base line BL, Fig. 1a, b), they received three 2 s foot shocks (unconditioned stimulus) in the conditioning context and 4 weeks later were re-exposed to the conditioned context for 3 min (Recall, Fig. 1a). The increased freezing during the memory recall session compared to baseline freezing levels indicated that fear memory was efficiently retained 1 month after conditioning (Fig. 1b). On the following day, animals were subjected to the extinction paradigm. Mice were put in the conditioned context twice per day for two extinction sessions of 3 min for 4 days. One day later (extinction memory, EM) and then again after 15 days (spontaneous recovery, SR), animals were re-exposed to the conditioned context for 3 min in order to test the persistence of fear attenuation obtained with this extinction protocol (Fig. 1a). This fear memory extinction paradigm led to a rapid decrease of freezing responses to the conditioned context that persisted for up to 2 weeks after the last extinction session (Fig. 1b).

In order to identify specific brain regions engaged during remote fear memory recall and attenuation, we compared animals subjected to the entire spaced extinction protocol (“Extinction” group), or to only the first recall session (“Recall” group), and to two control groups: a “Context Only” group, in which animals underwent the full spaced extinction but did not receive the foot shocks during fear conditioning; and a “Home Cage” group, where animals were subjected to spaced extinction, but their brain was collected after 24 h in the home cage. The “Context Only” control group was designed to correct for a possible unspecific brain activation induced by handling or by exposure to a familiar neutral context, while the “Home cage” control group accounted for possible persistent baseline activity changes induced by fear conditioning. Importantly, in the remote recall session, the “Home Cage,” “Recall,” and “Extinction” groups showed significantly increased freezing compared to the “Context Only” group, while, after the completion of the spaced extinction paradigm, the “Extinction” and “Home Cage” groups decreased freezing to a level comparable to that of animals of the “Context Only” group that never received a foot shock (Fig. 1d). In the “Context Only” group, we observed a slight increase in freezing between the first and the last exposure to the context (Fig. 1d, repeated measures ANOVA, *F*(1.7, 18) = 3.11, *P* = 0.075, *n* = 12), which could stem from incremental immobility deriving from a lack of exploratory drive to an already familiar context (Bernier et al. 2015, 2017).

Robust neuronal activation is followed by rapid induction of a number of activity-dependent genes known as immediate early genes. Among this class of genes, cFos expression peaks 90 min after neuronal activation and has been widely used to detect recently activated neurons (Douglas et al. 1988; Dragunow and Faull 1989; Guzowski et al. 2005). To map brain areas implicated in remote memory recall and attenuation, we collected brains from all four groups 90 min after the assigned behavioral session and performed cFos IHC on a selected set of cortical, thalamic, amygdalar, and hippocampal structures.

### cFos activation map of cortical structures

**Fig. 2:**
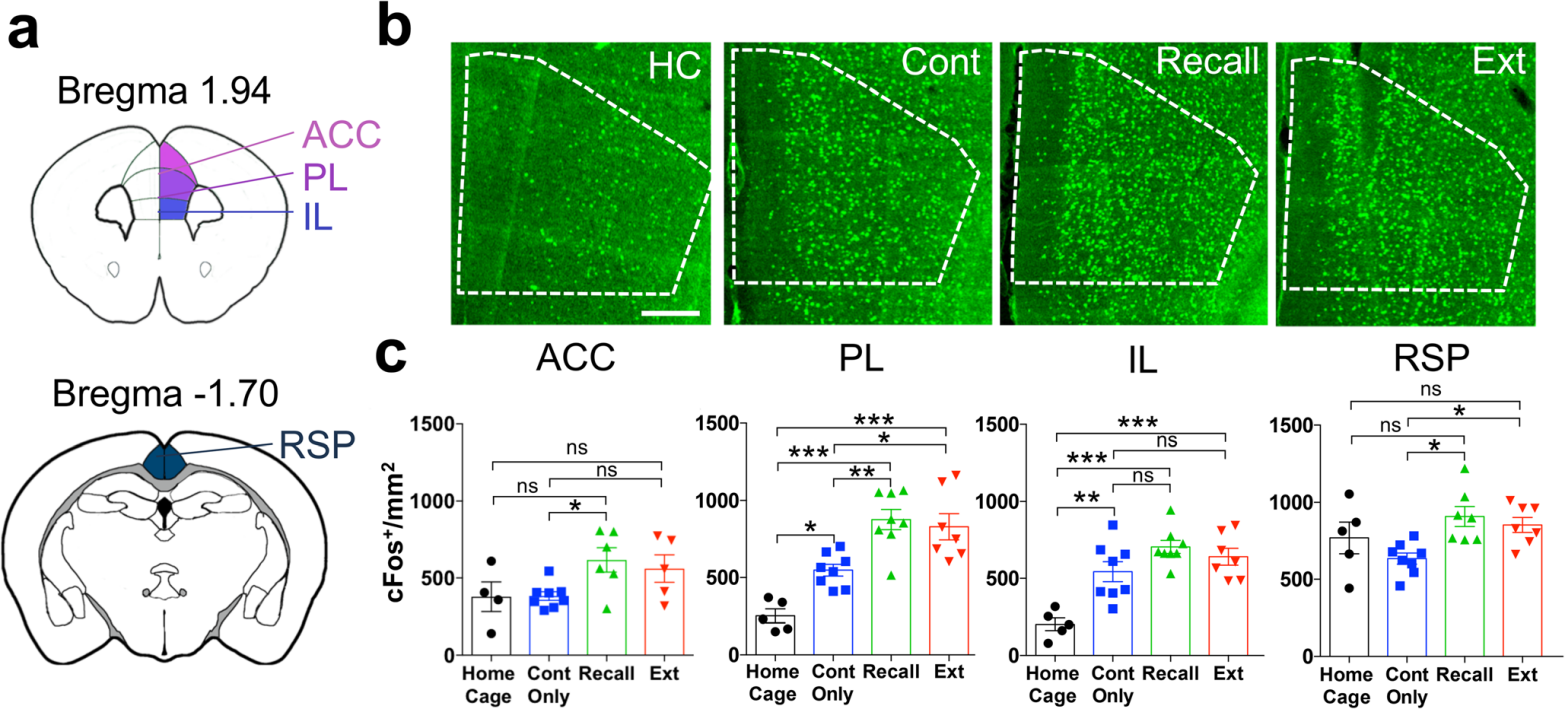
cFos activation in cortex. **a** Schematic representation of the cortical structures selected for cFos density analysis. **b** Representative pictures of cFos immunohistochemistry in the PL. Scale bar = 200 μm. **c** cFos density in “Home Cage,” “Context Only,” “Recall” and “Extinction” groups in the ACC (ANOVA, *F*(3, 24) = 3.3, *P* < 0.04, *n* = 5–8), PL (ANOVA, *F*(3, 24) = 18.1, *P* < 0.0001, *n* = 5–8), IL (ANOVA, *F*(3, 24) = 13.7, *P* < 0.0001, *n* = 5–8), and RSP (ANOVA, *F*(3, 24) = 4.4, *P* = 0.01, *n* = 5–8). *P < 0.05; **P < 0.01; ***P < 0.001, by Sidak post-hoc tests

The medial prefrontal cortex has been largely implicated in remote fear memory recall (Frankland et al. 2004; Corcoran et al. 2011; Einarsson and Nader 2012; Wheeler et al. 2013; Do-Monte et al. 2015). However, the involvement of its subregions in remote fear memory extinction remains elusive. While the roles of the infralimbic cortex (IL) and anterior cingulate cortex (ACC) were directly tested and showed controversial results (Vetere et al. 2012; Rosas-Vidal et al. 2014; Awad et al. 2015), activity in the prelimbic cortex (PL) was never directly assessed during remote fear memory extinction despite its well-established role in the fear memory circuit (Frankland and Bontempi 2005). Moreover, specific molecular changes within the retrosplenial cortex (RSP) have been linked to remote fear memory extinction (Corcoran et al. 2013). Based on this evidence, we chose the IL, PL, ACC, and RSP for cortical activity analysis (Fig. 2a).

All structures showed a significant increase of cFos positive cell density at remote recall compared to the “Context Only” group, except for the IL that showed increased cFos density at remote recall only in comparison to the “Home Cage” control (Fig. 2b, c). Increased cFos density compared to the “Home Cage” control was also observed in the IL and PL in the “Extinction” group (Fig. 2b, c). In the RSP and ACC, no significant differences to home cage baseline were found, probably because of the high degree of variability of this group (Fig. 2b, c). Interestingly, increased cFos was also observed in the “Context Only” group compared to “Home Cage” in the PL and IL, suggesting that these two structures may be recruited upon recall of a familiar neutral context (Fig 2b, c).

### cFos activation map of thalamic structures

**Fig. 3:**
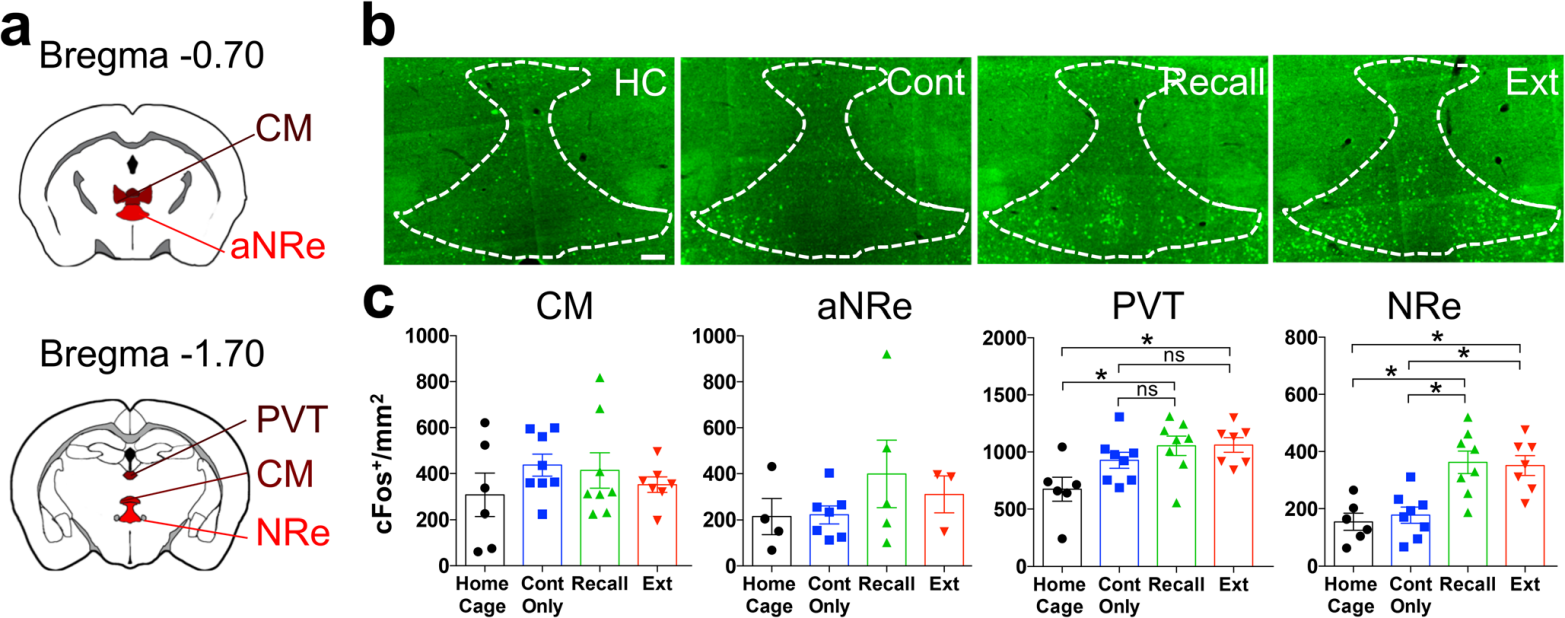
cFos activation in thalamus. **a** Schematic representation of the thalamic structures selected for cFos density analysis. **b** Representative pictures of cFos immunohistochemistry in the NRe. Scale bar = 100 μm. **c** cFos density in “Home Cage,” “Context Only,” “Recall,” and “Extinction” groups in the CM (ANOVA, *F*(3, 25) = 0.8, *P* = 0.5, *n* = 5–8), aNRe (ANOVA, *F*(3, 15) = 0.9, *P* < 0.4, *n* = 3–7), PVT (ANOVA, *F*(3, 25) = 4.4, *P* = 0.01, *n* = 6–8), and NRe (ANOVA, *F*(3, 25) = 10.5, *P* = 0.00001, *n* = 6–8). *P < 0.05; **P < 0.01; ***P < 0.001, by Sidak post-hoc tests

A growing body of studies has associated the midline thalamus with emotional memories (Wheeler et al. 2013; Do-Monte et al. 2015; Salay et al. 2018). In particular, the periventricular thalamus (PVT), and the nucleus reuniens of the thalamus (NRe) have been directly implicated in remote memories (Loureiro et al. 2012; Do-Monte et al. 2015), while the centromedial thalamus (CM) has only been investigated in recent fear memory extinction (Furlong et al. 2016). However, none of these nuclei has been investigated in relation to remote fear extinction. Here, we analyzed the CM, NRe, and PVT for cFos expression analysis (Fig. 3a). Of note, since the NRe and the rhomboid nucleus (Rh) are adjacent structures and have comparable connectivity and cell composition (Cassel et al. 2013), they were merged and further analyzed as one single area (NRe). In contrast, as the anterior and posterior subdivisions of the NRe have divergent outputs (Varela et al. 2014), the NRe was divided into its anterior (aNRe) and posterior subdivisions (NRe).

Analogously to previous reports, we observed increased cFos density in the PVT and NRe at remote recall (Fig 3b, c). Additionally, we found similar cFos levels at the end of extinction in these two structures suggesting that they may also contribute to this process (Fig 3b, c). Notably, only the posterior portion of the NRe showed activation at remote fear memory recall and extinction indicating that the anterior and posterior subregions of the nucleus reuniens may be functionally distinct. Similar to the PL and IL, the PVT showed a similar cFos increase in the “Context Only,” “Recall,” and “Extinction” groups compared to the “Home cage” group (Fig. 3c), suggesting that its activation may be related to the exposure to a familiar neutral context. No other midline thalamic nucleus showed any differences in cFos activity between the four experimental groups.

### cFos activation map of amygdalar structures

**Fig. 4:**
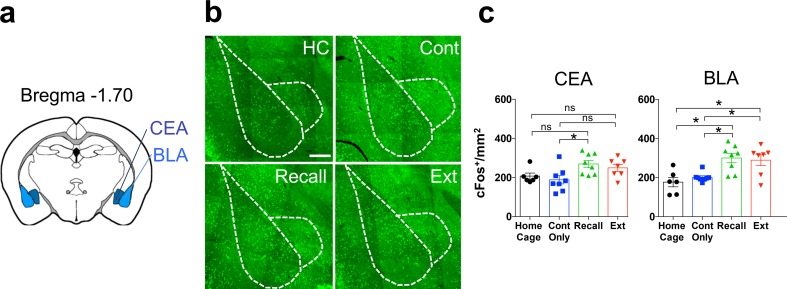
cFos activation in amygdala. **a** Schematic representation of the amygdalar structures selected for cFos density analysis. **b** Representative pictures of cFos immunohistochemistry in the BLA and CEA. Scale bar = 250 μm. **c** cFos density in “Home Cage,” “Context Only,” “Recall,” and “Extinction” groups in the CEA (ANOVA, *F*(3, 25) = 4.0, *P* = 0.01, *n* = 6–8) and BLA (ANOVA, *F*(3, 25) = 7.9, *P* = 0.0007, *n* = 6–8). *P < 0.05; **P < 0.01; ***P < 0.001, by Sidak post-hoc tests

The basolateral and central amygdala are considered key nodes in the encoding, recall, and extinction of recent fear memory (Trouche et al. 2013; Tovote et al. 2015; Silva et al. 2016). However, only a limited number of studies have implicated them in remote fear memory recall (Maren et al. 1996; Kitamura et al. 2017) and to our knowledge, no study has investigated the role of the amygdala during remote fear memory extinction. Therefore, we quantified cFos densities in the basolateral and central amygdala complexes (Fig. 4a).

While both structures showed a significant increase of cFos density at remote fear memory recall (Fig. 4b, c), with the CEA engaged to a lesser extent than the BLA, only the BLA showed increased cFos at remote fear memory extinction.

### cFos activation map of hippocampal structures

**Fig. 5:**
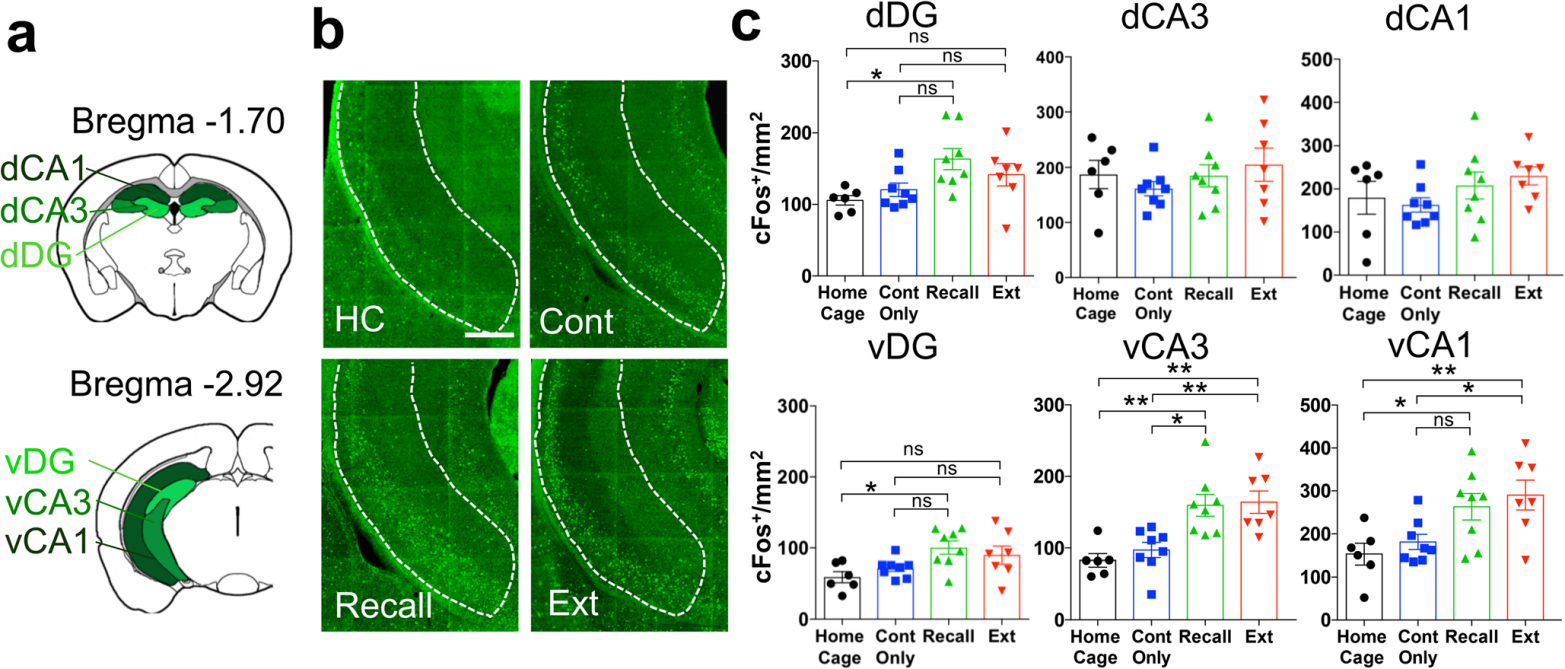
cFos activation in hippocampus. **a** Schematic representation of the hippocampal structures selected for cFos density analysis. **b** cFos density in “Home Cage,” “Context Only,” “Recall,” and “Extinction” groups in the dDG (ANOVA, *F*(3, 25) = 3.9, *P* = 0.02, *n* = 6–8) and dCA3 (ANOVA, *F*(3, 25) = 0.7, *P* = 0.5, *n* = 6–8), dCA1 (ANOVA, *F*(3, 25) = 1.2, *P* = 0.3, *n* = 6–8), vDG (ANOVA, *F*(3, 25) = 4.1, *P* = 0.02, *n* = 6–8), vCA3 (ANOVA, *F*(3, 25) = 9.6, *P* = 0.0002, *n* = 6–8) vCA1 (ANOVA, *F*(3, 25) = 5.2, *P* = 0.006, *n* = 6–8). *P < 0.05; **P < 0.01; ***P < 0.001, by Sidak post-hoc tests. **c** Representative pictures of cFos immunohistochemistry in the vCA1. Scale bar = 400 μm

One day-old fear memory recall heavily recruits the hippocampal formation, whereas remote recall induces much less IEG activation in this structure (Frankland et al. 2004; Maviel et al. 2004; Frankland and Bontempi 2005, but see Goshen et al. 2011; Tayler et al. 2013). However, to our knowledge, no study has tested IEG activation between remote recall and extinction of remote fear memories. In light of this, we examined cFos density of dorsal and ventral hippocampal subfields (Fig. 5a).

We found no cFos increase in the dorsal hippocampal subregions at remote recall (Fig. 5b). The same was true for the “Extinction” group, except for the dorsal dentate gyrus (dDG) which showed a higher cFos density at remote recall compared to the “Home cage” but not the “Context Only” control. The absence of dorsal hippocampal activation at both remote recall and extinction suggests that neither remote memory retrieval nor extinction of a consolidated memory recruits this structure. In contrast, both the ventral CA3 and CA1 showed significant cFos activity at remote recall and extinction, suggesting a functional dissociation of the dorsal and ventral hippocampal subfields for these timepoints (Fig. 5b, c).

### Interregional cFos correlations increase upon remote fear memory recall and extinction

**Fig. 6:**
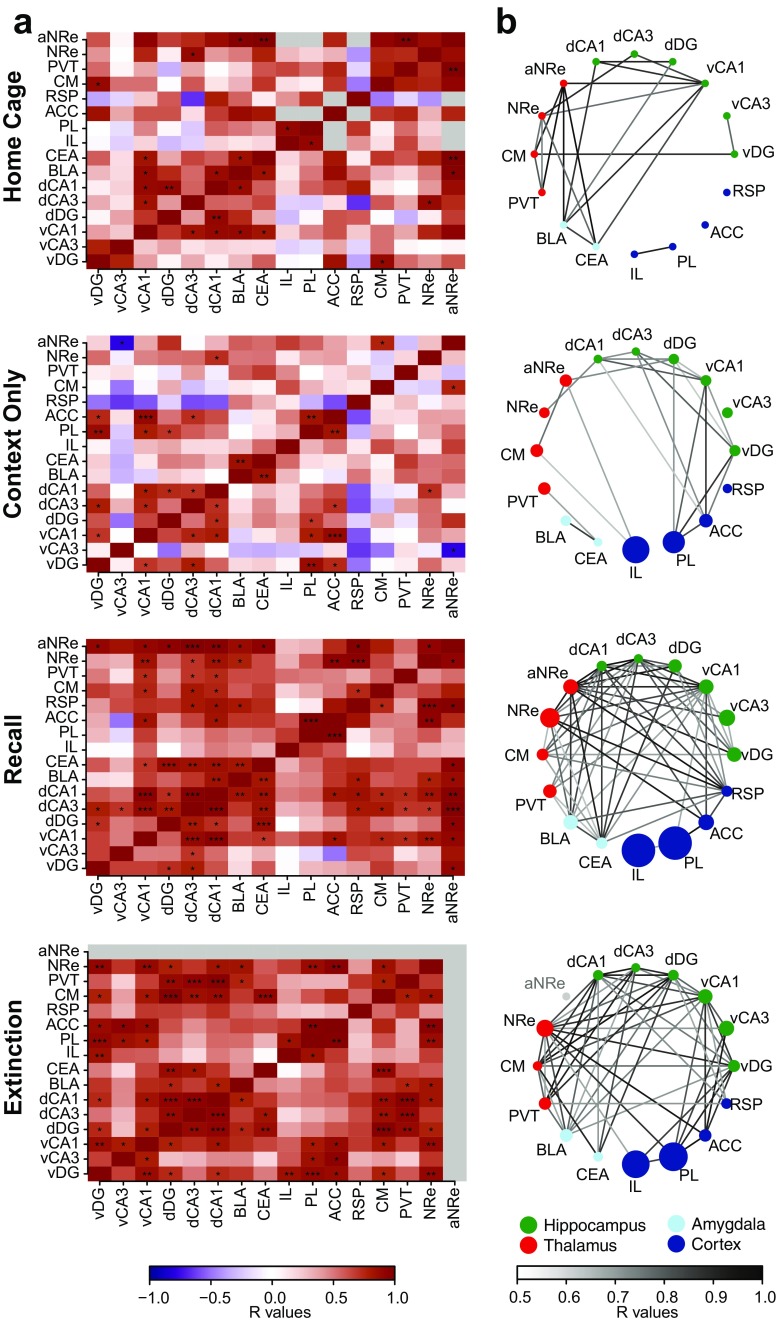
Cross-correlation and network connectivity analysis of cFos activationin in (from top to bottom) the “Home Cage”, “Context only”, “Recall” and “Extinction” groups. **a** Pearson correlation matrices showing inter-regional correlations for cFos activation density. Axes represent brain regions. Colors reflect Pearson correlation coefficients (scale, below) and labels within squares correspond to *P* values of correlations. *P < 0.05; **P < 0.01; ***P < 0.001. *R* values that were calculated using fewer than four pairs of cFos densities are shown as gray boxes. **b** Network connectivity graphs indicate only the strongest correlations (*r* value ≥ 0.5, *P* value ≤ 0.1, *n* value > 4). Connecting line transparency represents correlation strength (*r* value. Scale, below). Regions are color-grouped by major brain subdivisions and node size is proportional to the fold-change of cFos activation density between the indicated experiment and Home cage

In order to gain deeper insight into the functional connections within the set of forebrain structures identified by our analysis, we computed the covariance of each pair of regions across the subjects of each group. We first generated an inter-regional correlation matrix for each experimental group (Fig. 6a) which led to the identification of sets of regions whose cFos density co-varied across mice. Subsequently, we generated connectivity network graphs where only the strongest correlations were displayed (*r* value ≥ 0.5, *P* value ≤ 0.1, *n* value > 4, Fig. 6b). This analysis revealed a prominent increase of inter-regional correlations in the “Recall” and “Extinction” groups compared to control groups. Notably, apart from the high activity co-variance within the different hippocampal subfields, the most widely correlated structure in both experimental groups was the midline thalamus, particularly the NRe. This structure shows high cFos density correlations with cortical, hippocampal, and amygdalar regions. Importantly, these functional correlations reflect anatomical connections (Ohtake and Yamada 1989). Furthermore, both recall and extinction networks revealed high inter-regional functional connectivity even between structures that did not show cFos density increase, such as the dorsal hippocampus. This finding suggests that, for remote fear memory, the concerted activity of small subset of neurons within the different nodes of fear memory circuits may have a greater impact than the net activation of each structure alone. Finally, we found an extinction specific network correlation signature with high activity correlation between the ventral hippocampus and the medial prefrontal cortex that was absent in the other groups.

## Discussion

Here, we used cFos IHC to identify a set of forebrain regions recruited upon recall or extinction of a 1-month-old contextual fear memory in a novel spaced extinction test that mediates effective fear attenuation. Thereby, we generated, for the first time, an activation map of remote fear memory attenuation in comparison to remote recall across a set of selected cortical, thalamic, amygdalar, and hippocampal structures. The neural network composed of the prefrontal cortex, the amygdala, and the hippocampus has been widely studied for 1-day-old contextual fear memory recall and extinction (Bouton 2004; Myers and Davis 2007; Ehrlich et al. 2009; Herry et al. 2010; Johansen et al. 2011; Herry and Johansen 2014). At this time, a functional dissociation within this circuit has been observed, with hippocampal and amygdalar structures being crucial for memory recall and amygdalar and cortical structures representing predominant nodes for extinction (Orsini and Maren 2012; Vetere et al. 2012). However, at remote time points, when contextual fear memory recall is thought to be less dependent on the hippocampus and more dependent on cortical circuits (Frankland and Bontempi 2005), such dissociation has not yet been investigated. In the following sections, we discuss our results for each of the analyzed brain regions with respect to the knowledge gained from recent fear memory recall and extinction, before entering a more general discussion.

### Cortical structures

In line with the theory of system consolidation (Squire and Alvarez 1995; Dudai 2004; Frankland et al. 2004; Frankland and Bontempi 2005), we found a more pronounced recruitment of cortical than hippocampal structures upon remote fear memory recall (Figs. 2 and 5). Similar to previous studies (Frankland et al. 2004; Corcoran et al. 2011; Einarsson and Nader 2012; Do-Monte et al. 2015), we detected increased activity in the ACC, PL, and RSP cortices (Fig. 2b, c). In contrast, we only found increased activity in the IL in the recall group compared to home cage controls but not to non-shocked control animals. This discrepancy with previous findings, where the IL showed cFos induction upon remote recall compared to non-shocked animals (Wheeler et al. 2013), may, however, be explained by the slight differences in the behavioral features of the control groups. In particular, in our study “Context Only” animals were exposed to the conditioning context multiple times which may induce an association to this neutral context, as reflected by IL activation.

For remote memory extinction, we observed higher activity for RSP and PL cortices as compared to the context only group (Fig. 2b, c). The RSP has been shown to specifically mediate remote but not recent fear memory extinction via a NR2B/PKA/pCREB pathway (Corcoran et al. 2013) that is likely to be reflected in the cFos increase we observed (Flavell and Greenberg 2008). The PL has not been investigated in relation to remote memory extinction, but at recent time points its activity has been classically related to fear expression rather fear extinction (Milad and Quirk 2002). However, recent evidence showed that the PL also contributes to recent fear memory extinction through its projections to the IL (Marek et al. 2018). Our study suggests that a similar mechanism may play a role during remote extinction, since elevated PL activation was highly correlated with IL activity at remote extinction but not at remote recall (Fig. 6). Together, these lines of evidence stipulate that the PL may be engaged in different functional networks upon fear recall and extinction.

In our study, the ACC and IL did not show high cFos activity upon remote fear memory extinction. For the ACC, a previous study has reported similar results (Vetere et al. 2012). In contrast, the IL has been implicated in the extinction of 1-day-old fear memory (Orsini and Maren 2012), but its involvement in remote fear memory extinction is less clear. While Vetere et al. 2012 found no significant cFos increase in the IL upon remote fear memory extinction in comparison to pseudo-conditioned animals, selective functional manipulation of this region impaired remote fear memory extinction (Rosas-Vidal et al. 2014; Awad et al. 2015). Here, we observed no differences between cFos density in the “Context Only” and “Extinction” groups; however, both groups displayed higher cFos activation in comparison to “Home Cage” controls. This suggests that the activity in the IL may be important to recognize the contextual component of a remote memory independently of its emotional connotation and that its inhibition may therefore impair the updating of such memory. In line with this hypothesis, we found high activity correlations in a network including the IL, the ventral hippocampus, and the PL at remote extinction (Fig. 6a, b) that were absent at remote recall, corroborating the assumption that, despite the lack of cFos increase, the IL may play an active role in the extinction network.

### Thalamic nuclei

The midline thalamic nuclei, including the PVT, CM, and NRe—constituting the so-called limbic thalamus (Vertes et al. 2015)—are neuroanatomically favorably positioned to orchestrate recall and extinction of fear memory because they form a relay between the medial cortical, hippocampal and amygdalar regions (Eleore et al. 2011; Varela et al. 2014; Corcoran et al. 2016; Ferraris et al. 2018). We found a prominent cFos increase at remote recall and extinction in the NRe (Fig. 3c), which also emerged as an important hub of the remote recall and extinction correlation networks (Fig. 6). This finding is similar to previous reports implicating the NRe selectively in remote but not recent spatial (Loureiro et al. 2012) and remote contextual fear memory recall (Wheeler et al. 2013). Notably, the high activity pattern was specific for the posterior part of the NRe (Fig. 3c), suggesting that its antero-posterior portions may have dissimilar functions and may need be analyzed separately in further studies.

In the PVT, we found a remote recall and extinction-specific increase in cFos density compared to home cage controls, but not to the “Context Only” ones (Fig. 3c). This finding is different from a previous study, where the PVT was found to be involved in remote auditory fear memory recall (Do-Monte et al. 2015). Nevertheless, network analysis revealed high activity correlation of the PVT with amygdalar and hippocampal structures upon remote recall and extinction (Fig. 6b), corroborating the hypothesis that, despite the lack of specific activity increase, it may play an active role in both networks.

No activity changes were observed in the CM (Fig. 3c), which has been involved in 1-day-old cued fear memory extinction (Furlong et al. 2016). This finding may suggest that this structure plays a selective role for recent but not remote fear memory extinction, although the differential activation may also be explained by cued vs contextual conditioning strategies.

### Amygdalar regions

Amygdalar activation and plasticity are well known to contribute to 1-day-old fear memory extinction in humans and rodents (for reviews see Phelps et al. 2004; Herry et al. 2010). Additionally, the BLA has been recently associated to remote fear memory recall (Goshen et al. 2011; Do-Monte et al. 2015; Kitamura et al. 2017). In line with these findings, we observed increased cFos density in the BLA and CEA upon remote fear memory recall (Fig. 4b, c). Additionally, we found that remote memory extinction also induced a significant cFos increase in the BLA (Fig. 4b). For 1-day-old fear memory, the BLA has been shown to modulate fear expression and extinction through two distinct neuronal populations (Lee et al. 2013), therefore our BLA cFos induction results may point to a similar intra-amygdalar functional dissection for recall and extinction of consolidated fear memories. However, our network correlation analysis did not recapitulate the BLA connectivity data from 1-day-old fear memory studies, which showed that fear and extinction neurons in the BLA complex are modulated by the PL and IL respectively (Herry and Mons 2004; Herry et al. 2008). Here, we found low activity correlation between the BLA and the mPFC, while activity correlations were elevated with the midline thalamus and the dorsal and ventral hippocampi (Fig. 6), suggesting that the neuronal ensembles in the BLA may engage extinction brain networks differently before and after remote memory consolidation.

In line with previous studies on recent fear extinction (Lee et al. 2013; Furlong et al. 2016), we found no cFos increase upon remote fear memory extinction in the CEA suggesting that the CEA is preferentially recruited by high fear conditions, such as fear memory recall, while the BLA plays a more associative role required to modulate responses to both recall and extinction-associated contexts.

### Hippocampal regions

Even if a spatiotemporal shift from a hippocampus to a cortex-centered storage of conditioned fear memory during memory consolidation has been postulated (Frankland and Bontempi 2005; Wheeler et al. 2013), a growing body of evidence suggests that remote fear memory retrieval is not hippocampus-independent (Debiec et al. 2002; Goshen et al. 2011; Tayler et al. 2013; Gräff et al. 2014; Kitamura et al. 2017; Khalaf et al. 2018). For example, Goshen et al. found that, despite a non-significant cFos increase at remote memory recall, fear expression was impaired by a temporally precise inhibition of the hippocampal field dorsal CA1. In our study, at remote recall we also observed a negligible increase of cFos levels in the dorsal hippocampus (Fig 5c), which nonetheless correlated with cFos levels in the amygdala and ACC (Fig. 6). This finding thus corroborates the assumption of an efficient and sparse coding of remote memory in the hippocampus (Goshen et al. 2011).

On the other hand, for the ventral hippocampal fields vCA1 and vCA3, we found that both were engaged upon remote extinction to a similar extent as upon remote recall (Fig. 5b, c).

Interestingly, activity correlation analysis revealed a differential engagement of the ventral hippocampus in the extinction network with a strong functional connection with the mPFC that was absent in the recall network (Fig. 6). This evidence suggests that activity in the ventral hippocampus may serve a different function upon remote extinction compared to remote recall, such as an efficient updating of remote fear memories via a specific interplay with the mPFC. In line with this view, Gräff et al. (2014) showed that artificial induction of hippocampal plasticity in both CA1 and ACC was associated with remote fear memory attenuation. Alternatively, the differential cortico-hippocampal interplay between recall and extinction of remote memory could reflect a differential recruitment of the attention and behavioral flexibility brain systems that are known to reside in prefrontal cortical structures (Delatour and Gisquet-Verrier 2000).

## Conclusions and future directions

In sum, we found that both recall and extinction of a 1-month-old contextual fear memory recruit overlapping structures within the cortex, amygdala, and hippocampus. Likewise, we found a similar overlapping activation in a set of midline thalamic nuclei, a region that has recently been associated with remote fear memory recall (Wheeler et al. 2013; Do-Monte et al. 2015). Conversely, the exposure to a familiar neutral context (“Context only” group) did not induce activation in these areas, despite showing the same freezing levels as the “Extinction group.” Although other brain structures—not analyzed here—may also play an important role for either remote fear recall and/or extinction, these results indicate that upon remote fear memory attenuation, the brain activation pattern does not revert to a pre-training state but remains elevated.

This observation may reflect two scenarios. First, that activation in the fear extinction network is required to actively suppress fear. This assumption would therefore favor the view that extinction is a new learning process, during which a new trace of safety associated with the conditioned context is created (Bouton 2004; Myers and Davis 2007) and that this trace necessarily locates in the same structures as engaged in fear recall. Alternatively, the second scenario would be that the same network engaged upon recalling the original fear memory, i.e., the original memory trace of fear, has been updated towards safety during the extinction paradigm, and for this needs to remain active (Khalaf et al. 2018). This process is known as reconsolidation-updating (Tronson and Taylor 2007; Monfils et al. 2009; Lee et al. 2017). Since the extinction paradigm employed here consisted of two extinction trials per day, spaced by 2 h, which implies that the second trial is within the reconsolidation window of 6 h (Monfils et al. 2009), and since cFos was found to be overexpressed in conditions favoring the updating of remote contextual fear memories (Gräff et al. 2014), it is tempting to speculate that the similarity of the activation pattern between remote memory recall and extinction represents a physiological representation of reconsolidation. In line, our cross-correlation analysis revealed high functional connection between the ventral hippocampus and cortical areas at remote extinction that was absent at remote recall (Fig. 6), suggesting that the reverse emotional salience of fear extinction and recall may be underlined by a different network state rather than network structure. To address this issue, it would be important to determine whether, within the same set of brain areas, the same neuronal ensembles remain active from recall to extinction, and if the cFos densities of these two conditions are composed by overlapping or distinct fear and extinction neuronal ensembles (Herry et al. 2008; Khalaf et al. 2018).

Lastly, it is also important to keep in mind that the use of cFos to infer neural activity associations with discrete cognitive functions presents some potential limitations. First, its promoter may not be uniformly efficient throughout different brain structures and cell types and therefore special attention should be paid when directly comparing different regions. Second, its modest temporal resolution does not allow to account for differences between events occurring close in time (Guzowski et al. 2005). For example, we cannot account for activity differences occurring within each tested behavioral session, when low and high fear bouts may be intermingled. Third, since cFos basal expression levels are relatively low, it is not well suited to detect neuronal activity suppression, which also plays a crucial role in memory attenuation processes (Trouche et al. 2013).

These considerations notwithstanding, the results presented here help to shed light on the neural mechanisms underlying remote fear memory attenuation. Since fear recall and extinction rely on highly conserved circuits between rodents and humans (Hartley and Phelps 2010), these results may ultimately also contribute to identify efficient strategies to attenuate traumatic memories in stress and anxiety disorders such as PTSD.
